# The mule on the Mount Wilson trail: George Ellery Hale, American scientific cosmology, and cosmologies of American science

**DOI:** 10.1177/00732753231179330

**Published:** 2023-07-06

**Authors:** Kendrick Oliver

**Affiliations:** University of Southampton, UK

**Keywords:** George Ellery Hale, Walter Adams, Edwin Hubble, Mount Wilson Observatory, Pasadena, scientific cosmology, expanding universe

## Abstract

This article explores the relation between two different modes of cosmology: the social and the scientific. Over the twentieth century, scientific understandings of the dimensions and operations of the physical universe changed dramatically, significantly prompted by astronomical and astrophysical research undertaken at the Mount Wilson Observatory in Pasadena, California. Could those understandings be readily translated into social theory? Studies across a range of disciplines have intimated that the scientific cosmos might be less essential to the worlds of meaning and belonging that people and communities compose around themselves than more local and relational models of an ordered whole. The article applies that proposition to the Mount Wilson Observatory itself, arguing that the observatory’s founder, George Ellery Hale, and his acolytes were deeply invested in practices of terrestrial place-making, the politics of belonging, and the cadences of civilizational time as applied to their city and its region. Moreover, they struggled to construct a philosophy integrating the cosmos they were seeking to fix at home with the contortions and careering trajectories of the universal whole.

## Introduction

One day in June 1907, Walter Adams, a young astronomer working at the Mount Wilson Solar Observatory in Pasadena, California, received a telegram from a train on the Santa Fé railroad traveling southwest out of Chicago.^1^ The telegram announced that the most famous scientist in America, Professor Simon Newcomb, would be arriving in Pasadena the following morning and wished to visit the observatory’s facilities at the top of Mount Wilson.^2^ Adams hastened to make arrangements, securing two mules to convey Newcomb and himself up the steep switch-backed eight-and-a-half-mile mountain trail. After meeting Newcomb at a downtown streetcar stop, Adams took him to a corral at the bottom of the trail to saddle and mount his mule. By the way Newcomb looked at the animal, Adams guessed that he was unfamiliar with such a mode of transport, and so it proved. About half a mile along the trail, Newcomb’s mule – held with too loose a rein – decided to return to the corral, and Newcomb could not stop him. Persuaded at length to set out again, Newcomb insisted on holding an umbrella to protect his head from the sun; the umbrella kept catching in brush, agitating the mule and causing him to jerk precipitously close to the edge of the trail. Newcomb was also troubled by the prospect of encountering bears on the mountain. “All these factors,” Adams later observed, “tended to emphasize certain characteristics of Newcomb wherein he differed from the Christian saints.” By the end of the trek, “he was in a state of mind to make a dinner upon a menu of wire nails.”^3^ The next day, Newcomb elected to begin his descent down the mountain powered by his own two legs.

By the time Adams committed this story to print four decades later, the Mount Wilson Observatory could no longer be regarded as a remote, rustic outpost of American science, visited only under sufferance or by the most venturesome researchers. Between the wars, its astronomers and instruments transformed understandings of the physical cosmos, drawing scientific talent and ideas about the careers of the stars, the galaxies, and the wider universe into circuits around itself, securing the observatory’s reputation as the leading astronomical institution in the world. But the tale of Newcomb’s fretful, stuttering progress up the Mount Wilson trail captures the tensions that were already creatively reshaping American astronomy as the observatory – established only three years before – consolidated its claim upon the mountain.

To build an observatory in such a place was not just to reconfigure the geography of the discipline, projecting a new salient out, simultaneously, to the south, to the west, and up into the sky; it was to propose a challenge to standard astronomical practices and – for a while at least – to the cultural identity of the American astronomer as well. Newcomb was a grandee of the discipline’s positional and astrometric traditions, which prioritized the tasks of precisely cataloguing the locations of celestial objects and calculating their motions over time. Like many positional astronomers, Newcomb, as the former superintendent of the Nautical Almanac Office, had labored within the precincts of the nation state and attended closely to its instrumental needs; he also participated enthusiastically in the transnational program of systemizing methods and standardizing measures, sharing in the consensus that the astronomer’s most essential tool was a refracting telescope, which, with a sturdy mount, could be reliably directed from star to star over the course of a night’s seeing, determining the coordinates of each in turn.^4^

When he traveled out to California and climbed up Mount Wilson, however, Newcomb encountered an astronomical milieu that was very different from his own. Mount Wilson astronomers, supported by philanthropy rather than the state, enjoyed a dispensation to move beyond the more obviously “useful art” of logging the positions of stars by making inquiries into the physical and chemical processes that caused them to be born, shine brightly, and die. In order to carry out this “new astronomy,” the observatory looked to provision itself with an array of innovative instruments: a large reflecting telescope that could gather all the light of a single celestial object on its mirror for hours at a time; spectroscopes to disaggregate that light by wavelength to discern the object’s chemical composition; and cameras capable of capturing long exposures, including spectrographic exposures, that would continue to be available for analysis after the original observation was complete.^5^ Hence, also, the requirement that the observatory’s telescopes be sited far away from competing sources of light and at an altitude that reduced atmospheric disturbance and dispersion, without being so remote that construction and effective maintenance were impracticable. That is, the “new astronomy” was best conducted in locations, such as the summit of Mount Wilson, that positional astronomers were not used to frequenting, locations that represented the keenest frontier between the natural world and scientific modernity on the mainland United States, locations serviced by creatures like the mule, for whom human intentions and directions were necessarily subordinate to the rule of their own instincts. For Adams, it seems, Newcomb’s unwillingness to adapt himself to his mule was of a piece with his indifferent response to the instruments of the “new astronomy” when he finally reached the Mount Wilson Observatory and toured its facilities: “the spectrographs and spectroheliographs were not merely a closed book to his mind but one which ought to remain closed.”^6^ Both on the trail and at the top of the mountain, the promise of a novel, fulfilling encounter with physical nature had withered in the chill of Newcomb’s insensibility and disdain.

Some of the inferences invited by Adams’ account of Newcomb’s visit may have been unjust to positional astronomers, many of whom did readily depart metropolitan centers to seek alternative points of vantage upon the skies, and to Newcomb personally, for he was not antipathetic to mountain observatories per se.^7^ But the story illuminates the intricate webs of association that could be spun between particular modes of astronomical inquiry, the different environments in which they tended to be practiced, and the personal characteristics that such environments drew out in their resident astronomers. As Maria Lane demonstrates in her study of the role played by mountain observatories in debates about Martian geography, these webs of association were also matrices of value.^8^ For its founding astronomers, Mount Wilson – the observatory, but also the mountain – was *the* place to be, better than almost anywhere else. In time, the mountain would offer the world the best perspective available on the large-scale dynamics of the physical universe, but it represented for those founders, as they kindled the observatory into life, something of a cosmos in and of itself: a distinct, bounded space attended by common rituals of necessity, shared aspirations, and a praxis of consideration, interest, and affection bonding the astronomers to one another, to the landscape around them, and to many of its other animal inhabitants, mules included.

Not much more than a decade later, however, and the contours and conventions of the world that the astronomers had built for themselves on Mount Wilson started to flex and change, just as their observations would, in due course, extend the boundaries of the Milky Way and then enfold it within a universe astir with other galaxies and still expanding in size.^9^ The observatory’s facilities on the mountain would become more accessible; the work routines of its staff would cease to evoke the exigencies of frontier life, reflecting an absorption into the syncopated rhythms of trade, clerical labor, and professional life pacing the experience of time at the foot of the mountain, in downtown Pasadena; and the ambitions of the observatory’s senior personnel would be directed across broader earthly as well as celestial horizons, encompassing a vision of Pasadena as a new scientific and cultural metropole of “Anglo-Saxon” civilization. By the mid-1920s, Mount Wilson was no longer a world unto itself, but it continued to inspire practices of place-making that seeded new institutions in service of a theory of history, embedded hierarchies of belonging within those institutions and the spatial environment around them, and embodied the promise of effective agency against the specter of decadence and decline. The mountain functioned as a fulcrum for two models of the ordered whole – one scientific and oriented to the universe at large, the other local and relational, invested in Pasadena as a hearth and home.

This article examines the efforts of George Ellery Hale, Mount Wilson’s founding director, to orchestrate space and social relations in and around the observatory in conformance with a presumption that the capacity to contribute to the advance of science and to the cultural elevation of a modern American city lay, almost exclusively, in the possession of white men of his own class. It also explores the salience of such efforts to Hale’s role as a science communicator, composing popular accounts of the “new heavens” that Mount Wilson’s telescopes were bringing into view. In particular, the article identifies a striking contrast between Hale’s assiduousness in applying his social theories to his own local environment and the hesitation with which, in his writings, he addressed the philosophical and social implications of an expanded, and expanding, universe. In that contrast, it suggests, lies a clue of some significance. According to John Tresch, historians of science have for some decades distrusted the proposition that all lines of investigation into the natural world necessarily flow out into the grand oceanic commons of cosmological inquiry. Instead, they have situated their protagonists within specific “cartographies of knowledge” – zones, networks, systems – substantiated around material objects, practices, and institutions; these were not settings from which such protagonists might reflexively command and explain a universal whole.^10^ Yet, as Tresch observes, “locally situated acts and objects” may still speak of a cosmology, disclosing assumptions about the origins, trajectories, and essences of an encompassing world, even if those assumptions, “riddled with gaps and counterpoints,” are never ultimately resolved into a coherent conceptual scheme.^11^ Tresch challenges historians of science to attend to the “realist force” of the cosmological ideas that “arise from and feed back into” scientific work, whether or not that work directly essays the immensities of cosmic space and time.

This article takes seriously the question of how scientific workers situated themselves in relation to a cosmology, but it also identifies Tresch’s “gaps and counterpoints” as meaningful signals in themselves, disclosing the presence of discontinuities and zones of disarrangement between the immediate, everyday settings of human thought and the amplitudes of the astronomical universe. Although texts describing the advance of the space sciences and space technologies still frequently invoke, as if by rote, the astrofuturist premise of an innate, instinctual human orientation to the skies, it is possible to collate from disparate bays of our libraries an alternative corpus of sorts: one that correlates accounts of philosophical estrangement from the cosmos in the wake of the scientific revolution with works that survey and/or endorse attitudes of skepticism and indifference toward the goals of the space age, that reflect on the continued tug of terrestrial mass and matter upon social constructions of nature, that cast the experience of dwelling in place *on* the earth, *under* the sky, as the elemental source of humans’ sense of being. Across this corpus, the contents and dimensions of the modern physical universe appear as unassimilable to and incommensurate with human needs.

For Alexandre Koyré, Michel Blay, and Rémi Brague, the spatial hierarchies of the medieval cosmos, which folded mankind within a circumference of providential care and offered natural philosophers an index to the whole of creation, were dissolved by “the moderns”: Cartesian and Newtonian physics distended the universe toward the infinite, exceeding the reach of human reason, and placed it under the governance of forces that were blind to mankind.^12^ Apprehensions of an indifferent universe, and the collateral conviction that the destiny of the human species remained tied to the fate of the earth, were – against some early expectations – reaffirmed even as the rockets of major nations achieved escape velocity and dispatched probes into space.^13^ The Apollo program only rarely received majority support from the American public, and it was not the samples of moon rocks collected on its missions that most conspicuously decorated space age debates about the future of humanity, but rather the images that astronauts had taken of their home planet, liquid, alive, and alone.^14^ Into the present, and an accelerating climate catastrophe – as Bruno Latour noted – refutes any conception of humankind as separate from nature and compels a politics directed away from outer space and back toward the defense of “that without which you would not be able to live”: that is, a habitable earth.^15^

Although cosmology as a scientific field has come increasingly to contest the grounds for claiming a special physical status for the earth, the worlds that cultures and movements compose around themselves have continued to retain the planet or – more recently – the “globe” as a critical reference frame: large enough to intimate that their perspectives are not provincial, but more legible and inspirited than the fathomless void beyond.^16^ Yet such worlds may also be composed upon terrains much less extensive in their circumference than the whole of the earth: measured out by no more than an ambling walk and a count of homes within easy sight, rather than in meridians and via nation-state census. Students of human society have revised the assumption of classical sociology that the “essential ideas” signified by a cosmology – about the organization of space, the operations of time, humanity’s place within nature, and the order of interhuman relations – must be commonly held across a culture.^17^ The sociology of everyday life – though still attentive to the governing power of collective norms – has credited individual experience and microsocial interactions with significant agency in the process by which people come to inhabit a meaningful cosmos.^18^ The reassurance that we live out our days within a meaningful order may derive – as ethnographers have noted – from practices and experiences that are embodied, grounded, local, and relational, as much as from impressions of the cosmic or planetary whole.^19^ Meanwhile, the turn of the postmodern humanities to a re-engagement with the thought of Martin Heidegger has left a discernible heelmark even within texts on the space age and the space sciences.^20^ Heidegger’s emphasis upon the human need for an experience of dwelling, incubated in spaces “through which we go daily,” attended by “ready-to-hand” objects that involve us in our immediate world, challenged the modernist assumption that advanced technology, globalized politics, and astronomical soundings of the extragalactic universe would necessarily elevate the moral and philosophical condition of mankind.^21^ But, as Heidegger’s commitments to Nazism attest, cosmologies that associate being with belonging-in-place – that qualify personhood by reference to the possession of space, and the possession of space by reference to theories of nativity – themselves provide a script for deviations away from humane perspective and humanitarian policy, legitimizing regimes of exclusion and violation: the local worlds defined by such cosmologies are for some and not for all.^22^

In the first decades of the twentieth century, the roiling universe unveiled by the synthesis of general relativity and nuclear physics had not yet come fully into focus, even for those who surveyed it every day. Astronomers – especially at Mount Wilson – were starting to comprehend the lifecycle of stars, reckon interstellar distances, discern the chemical signatures that revealed the motion and velocity of remote celestial objects, and conjure warily with the contortions and curves of relativistic space. It was not unreasonable for non-astronomers, their attention snagged by press reports of the latest discoveries, to wonder what sort of philosophies they affirmed, directly or via analogy.^23^ But astronomers, even those – like Hale – who were comfortable writing for a popular audience, were, on the whole, hesitant to venture detailed thoughts on the matter. This article proposes that such hesitation involved something other than a fastidious concern with holding the academic discipline of science aloof from the more speculative trade of fabricating social theory. Mount Wilson’s astronomers were deeply invested in the social worlds around them. Both in its early years, as it was sinking its foundations into the mountain, and later, when its achievements were studding Pasadena onto maps of nation and globe, the observatory was a crucible of principles and practices with significant social implications. But those principles and practices were not derived substantially from contemplations of the starry skies in all their spatial and temporal breadth, because such contemplations provided few compelling prompts to an operative philosophy. Instead, Hale’s cosmology was keyed to the power of particular institutions, places, and social groups to command their own earthly present and the time next to come.

## Mount Wilson as a cosmos

The proposition that the efforts of scientific cosmology to fathom the deepest expanses of time and space have been conditioned by more proximate meridians of social experience is, of course, consistent with the premises of the sociology of scientific knowledge since the 1970s and much recent research in the history of science.^24^ The attention that scholars have directed toward the action of locality, place, and the built environment upon the organization and outcomes of scientific work, and their concomitant concern with how science attains authority precisely by blurring the imprint of specific geographical contexts upon its operations, may yield particular dividends when applied to the field of cosmology.^25^ As Lane notes, it was not just a desire to minimize the atmospheric distortion of starlight that motivated astronomers in the nineteenth and early twentieth centuries to haul their telescopes to the tops of mountains, and sometimes thereafter to establish permanent high-altitude observatories. The Romantic movement, in combination with stratigraphic geology, had consecrated mountains as the commanding seats of perspective into both the interminable layers of earthly time and the infinite reach of the heavens, while the exacting character of the sierran terrain invited an association between the fortitude and rigor that was required to set up camp in such locations and the discipline – in both senses of the word – of the astronomer.^26^ Observations carried out on mountain summits enjoyed an elevated reputation, almost irrespective of whether they had been carried out well. In the otherness of these environments, and in the strange ascetic routines they adopted there, astronomers seemed rather like medieval monks redeployed to the enterprise of scientific revelation: here they were once more, transgressing circadian rhythms, attending closely to the passage of nocturnal time, communing with the heavens at a remove from the madding crowd. Hale, in Mount Wilson’s founding year, had deliberately invoked the analogy when he gave the name “the Monastery” to the building that would house the living quarters for astronomers conducting observations on the peak.^27^

In later years, as journeys to the mountain summit became easier and as the facilities there assumed the bearings of a major scientific installation, both Hale and Adams would wax nostalgic about the early days of the observatory, when its astronomers, by choice as well as necessity, led a more hermetic existence. Their memories, confirmed by correspondence at the time, attest to the affective investment they made in Mount Wilson, an investment attended by desire for renewals of self, for a sensualizing immersion in the natural world, and for homosocial intimacies of communication in an enclosed, monocultural space. Hale’s interest in establishing an observatory on the mountain had been shaped by a number of factors, including its potential as a site for a solar telescope, but it is evident that the sunlight and transparent air at its peak also beckoned him out of the fog of a profound personal malaise. Hale was director of the Yerkes Observatory, located in Williams Bay, Wisconsin, a lengthy commute from his family home in Chicago: the winters in Williams Bay bit hard, the skies were often shrouded in cloud, and Hale had been struggling to raise money to build an instrument – a sixty-inch reflecting telescope – that he regarded as essential to the progress of Yerkes and the astronomical discipline more generally. Following a short visit to Mount Wilson in the summer of 1903 to survey the atmospheric conditions, Hale returned to Pasadena in December. Disembarking from the train into the light and warmth of a summer’s day, his depression, he recalled, “quickly vanished.”^28^ His family, wintering with him, relished the climate of southern California, the trees around their bungalow lively with bird song and laden with fruit, but for Hale himself, the mountain was the thing. Donning the costume of “a California pioneer,” Hale made regular ascents up the Mount Wilson trail over the next four months; he hired mules to convey equipment and supplies to the top and, with the help of a carpenter and a mason, upgraded an abandoned log cabin named the Casino into a habitable dwelling; and when more supplies were required, he descended the mountain in the effervescent manner of youth: on his rear, via slides worn out between the zigzags of the trail.

Hale’s enthusiastic errand into the semi-wilderness was very much of the moment: in 1903, President Roosevelt, casting himself again as the American outdoorsman par excellence, had famously spent sixteen days camping in Yellowstone National Park and another three in Yosemite.^29^ For some of his colleagues back in Williams Bay, Hale’s letters from Pasadena, evoking the “exhilaration” of his work on Mount Wilson, kindled thoughts about the possibility of joining him there.^30^ Adams, describing his “general state of gloom” as an “exceptionally dreary” Wisconsin winter dragged on, informed Hale that the “prospects of a bohemian year on Mount Wilson with you appeal to me very strongly,” so strongly indeed that he was prepared to live without salary if that was required. He looked forward, at the very least, to spending the summer in California continuing Hale’s observations of the sun when Hale himself was likely to be needed back at Yerkes. Adams asked Hale to identify “a few trees” on the mountain that were well-suited for meditation, as he had “hopes by dint of sufficient self-communion and gazing off into space to be able to found a totally new system of philosophy in the course of the months there alone.”^31^

As it turned out, Adams did not have to sacrifice his salary to work at Mount Wilson, or subsist there in isolation for any period of time. In April 1904, the Carnegie Institution of Washington provided an initial grant to support the transfer of a large solar telescope from Yerkes, and in December it approved an appropriation that allowed Hale to establish a permanent observatory on the mountain, complete with a sixty-inch reflector.^32^ Hale recruited Adams and two of his Yerkes colleagues, Ferdinand Ellerman and George Ritchey, to join him in California. Decades later, Adams retained an acute memory of his journey south, traveling in the company of Hale and Ritchey, and his awareness as the train approached Pasadena “that a new life with new responsibilities and opportunities lay before us all.”^33^ Adams, Ellerman, and Hale spent much of the following year on Mount Wilson, supervising construction, setting up instruments, or conducting solar observations. In their remote encampment, they experienced relatively few human intrusions, though they warmly welcomed scientific visitors from elsewhere eager to see the “new astronomy” carried out so close to the sky. The mountain environment was vivid with wildlife; encounters with rattlesnakes, Adams noted, were “fairly common.” The astronomers developed an affection for the mules – each of them named and unique in temperament – that supplied the observatory with food, equipment, and materials every day. In the evenings, when there was no sun hanging in the heavens to hold them at work, the group would gather for dinner in the Casino or, when it was completed at the end of 1904, the Monastery. In those intimate spaces, around a log fire, they would discuss current affairs or listen to Hale’s accounts of his transactions with the great men of science and politics. One frequent guest, Charles Abbot of the Smithsonian Institution, would provide dramatic readings of an antisemitic tale describing the comeuppance of a deceitful Jewish trader in Constantinople, drawn from Robert Curzon’s *Visits to the Monasteries of the Levant*.^34^ In this period, in the tight little world they had made for themselves on the mountain, Hale and his acolytes enjoyed, Adams recalled, “many good times together.”^35^ His reminiscences – limpid, detailed, but brushed in sepia – suggest that they were never as happy again.

## Pasadena as a cosmos

The astronomers’ time as inhabitants of a mountain paradise came to an end by progressive degrees, as they themselves nudged apart its gates to address a larger cosmos, in the sky and across the horizons of human society below. Over the first ten years of its existence, the observatory’s investigations widened in scope, out from its original primary focus on the earth’s own sun to the evolution of stars in general, an interest that in turn directed attention to the problem of the spiral nebulae: were these mysterious clouds small nurseries in which individual stars spun into life, or massive star systems external to the Milky Way?^36^ In 1914, using the sixty-inch telescope, Adams developed a spectroscopic method for ascertaining the distance to remote stars, opening up the dimensional depths of the celestial canopy to astronomical measurement.^37^ The telescope also wrought a change in the operational radius of the observatory itself. To facilitate the construction of the telescope, complete with mounting and dome, the Mount Wilson trail was widened into a road.^38^ Although the observatory continued for a number of years to rely on daily pack trains for supply, the purchase of two trucks in 1912 finally made the mules redundant; from then on, astronomers could hitch a ride from Pasadena and reach the summit within two and a half hours, instead of having to trek for the best part of a day.^39^ As access to the summit improved, the number of visitors increased. Some of them were astronomers drawn from other institutions by the promise of seeing time on the sixty-inch telescope or by other research collaborations, but the opening of a hotel on the peak in 1906 required the observatory to adjust to the interest of lay sightseers, especially during the passage of Halley’s comet in May 1910.^40^ Meanwhile, the scale and exacting specifications of the sixty-inch telescope, and of the 100-inch instrument that Hale aimed to build thereafter, led Hale and his staff to turn over some large manufacturing tasks to outside contractors based in San Francisco, Boston, and Paris, and to spend time at those contractors monitoring their work.^41^ Adopting the principle that it would dedicate its site on Mount Wilson to the core function of stargazing, the observatory purchased a plot of land on Santa Barbara Street in downtown Pasadena, aided by a public subscription drive organized by the city’s Board of Trade, and by 1912, a substantial headquarters building occupied the plot, comprising offices, laboratories, a dark room, machine and instrument shops, and a library.^42^ The daily routines of Mount Wilson’s astronomers came to resemble those of other middle-class residents of Pasadena: the mountain called to them less often, and for shorter periods of time. Once the sixty-inch telescope entered operation at the end of 1908, the astronomers enjoyed fewer evenings gathered together convivially in front of the Monastery fireplace; they were either using the telescope to scan the night sky or eating supper at home.^43^

To the astronomer setting out on his hundredth commute up Mount Wilson, the summit continued to beckon a welcome into a distinct, charismatic space. An ascent that began in a murk of drizzle and mist might end above the cloud line bathed in brilliant light.^44^ In 1924, when Adams succeeded Hale as director, he was still able to describe the mountain as “almost a separate world.”^45^ This was an assertion made, however, in the context of correspondence about the observatory’s exemption from state taxes, as Adams weighed the value of its contribution to California’s educational economy against the limited benefits it derived, as a result of its remote location, from services funded out of the public purse. Adams had inherited from Hale a complex vision of the observatory as an institution that could stand aloof from the everyday scrum of worldly interests and concerns while consolidating its status as an integral node within networks of scientific movement and exchange and serving as a catalyst for regional transformation. As its director, Adams was obliged to represent the observatory as it developed its associations with the city of Pasadena and communities beyond, but it is evident that he did not enjoy all the political dimensions of this task. Adams had originally been drawn to scientific work out of an appreciation for order; science, he believed, addressed itself to realities that were fixed and enduring, or at least predictable, in contrast with the humanities and social sciences, whose subjects, changing ceaselessly, embarrassed any claim to have distilled their substance and action into fundamental truth.^46^ The chemistry of the stars could be fixed and comprehended through procedure; the strife and tumult of interwar Californian politics could not. In 1934, just as the socialist writer Upton Sinclair’s campaign to become governor was drawing fierce, paranoid fire from the right-wing press and business and religious groupings across the state, Adams told one correspondent that, though there were “many pleasant features” to life in California, “there is in Los Angeles, at least, a lack of stability in institutions under conditions of stress unless they have powerful political support.”^47^

Long before he was replaced by Adams as director of the observatory, Hale had ceased to be a regular presence on Mount Wilson. This was in part due to a recurrent mental illness, which waylaid him for more than a year in 1910–11 and continued, off and on, to haunt his health through the 1920s and 1930s.^48^ But it was also a consequence of his tendency to immerse himself in outside projects, which – in contrast to his successor – reliably excited within Hale an enthusiasm and a sense of purpose that, as time went on, his own astronomical research did not.^49^ Hale was a builder of things and a maker of places; as soon as he dropped anchor in a new setting, he began to imagine how he might change that setting – for the better, he believed. And so, much as he had relished the seclusion and pioneer stylings of the observatory in its first years of operation, the simple and immediate pleasures afforded by that romance yielded quite quickly to the seductive, enduring stimulus supplied by the orchestration of a broader strategic scheme, in which the observatory was a hermetic retreat no longer, serving instead as a kind of temple seated on a mount, inspiring, ordering, and civilizing the society around it.

In 1907, three years after his arrival in Pasadena, Hale became a trustee of the Throop Polytechnic Institute, a small, undistinguished vocational school in the city, and started to plot for it a path, involving the recruitment of talented leaders and tapping the beneficence of local elites, by which it could develop into an establishment that exemplified excellence in scientific and engineering education – as at length it did, under a new title, the California Institute of Technology.^50^ Around the same time, and with similar tenacity, Hale began to pay court to the railroad magnate Henry E. Huntington, who was developing an estate in San Marino, just south of Pasadena.^51^ Hale eventually persuaded Huntington to create and endow a research library “of the highest type” around his collection of rare books, manuscripts, and art, with the aim of attracting renowned scholars of the humanities to the area, just as astronomers, physicists, and chemists from “all parts of the world” were being drawn to Mount Wilson and Caltech.^52^ In a theme adapted from other regional boosters, who had confected a vision of southern California – through its synthesis of a Mediterranean climate, the spiritual and aesthetic inheritance of Spanish frontier missions, Yankee Protestant high-mindedness, and the productivity of migrated mid-Westerners – as the ideal setting for perfecting civilization, Hale was convinced that Pasadena, with its emerging reputation as a center of learning and culture, could achieve the status of a new Athens.^53^ He guided the efforts of city planners to furnish it with a civic architecture to match, resulting in the construction of a city hall, auditorium, and public library, though not, as Hale had once hoped, a perfect replica of the Parthenon; meanwhile, an Athenaeum club, named after the temple of Athena, the meeting-place of poets and philosophers, and modeled on the London institution where Hale had spent many happy hours conversing with his British scientific peers, was established on the Caltech campus.^54^

During the First World War, Hale relocated to Washington, DC, where – as chairman of the National Research Council – he directed the resources of American science to meet the technical needs of the armed services.^55^ As the end of the conflict approached, however, he sponsored the formation of an International Research Council, subsequently the International Council of Scientific Unions, to reopen the channels of transnational scientific communication and collaboration closed down by the war, carefully fending off the revanchist insistence of many Allied scientists that German researchers, even those who had played no part in the Kaiser’s military schemes, could never be welcomed back into the fold.^56^ In the years before the continent fell to arms, a number of European astronomers had visited the Mount Wilson Observatory, attending meetings, using its telescopes, and participating in joint research projects with its staff, and Hale was now keen to renew that welcome and to extend it to encompass Caltech, so that scientists across the world, across all the principal scientific fields, would look upon Pasadena as the intellectual metropolis where they could do their best work and live their best lives.

That aspiration was markedly advanced by Mount Wilson’s decisive interventions in two of the most conspicuous scientific debates of the 1920s. In 1924–5, Mount Wilson astronomer Edwin Hubble used the observatory’s new 100-inch reflector to derive the distances to two spiral nebulae – Andromeda and M33 – placing them both well beyond the boundaries of the Milky Way.^57^ Having established that many of the nebulae discernible in the heavens were extragalactic, Hubble then turned to observation of their motions, with the aid of a spectrograph attached to the 100-inch reflector. Hubble’s results – which revealed that the nebulae furthest in distance from earth were also moving away the most rapidly – suggested that the universe was in a state of expansion.^58^ It was a finding that moved Pasadena to the center of the international controversy surrounding Albert Einstein’s theory of general relativity.^59^ Hubble’s data was compatible with a relativistic universe, but not with the static version of it that Einstein had originally preferred. In 1931, Einstein himself traveled from Berlin for a two-month sojourn in Pasadena, where – with the regional press in constant attendance – he was transported up Mount Wilson to make observations of nebulae through the 100-inch, conferred with physicists and mathematicians at Caltech, and seemed to concede, on the basis of the new evidence, that his relativistic field equations required a non-static solution.^60^ Einstein also delivered a short talk, carried by radio across the nation, from a formal dinner held in his honor at the Athenaeum.^61^ In that moment, Hale’s ambitions – for Pasadena and its place within the international republic of science – appeared to have been fulfilled, though he was too fragile in health to personally participate in the festivities.^62^ Hale, observed one reporter during Einstein’s visit, “has been the single greatest factor in making Southern California a world center of science and of bringing [sic.] the rest of the universe vastly nearer to our own world, so near that no one can prophesy what astounding new era soon may dawn.”^63^

## Orders of belonging in Hale’s Pasadena

It is one of the functions of a cosmology to distinguish qualitatively between different places, to identify centers and peripheries and explain the status of each, including – occasionally – redistributions of status, when a site that was previously peripheral to a particular cultural system becomes endowed with the potential to renew or transform that system, or when a metropole succumbs to decadence.^64^ But cosmologies also work to order the mobility of people within places, especially places of importance, by defining a hierarchy of authority and belonging. They have often required, or justified a requirement for, a distinctive priestly class, the members of which claim an enhanced capacity to apprehend the nature of the universe and thereby also a privileged access to the sites of social power and the means of intellectual production.^65^ Admission to and movements through the spaces of scientific inquiry have been regulated according to a similar caste structure, a resemblance attributable in part to the enduring imprint of religious tradition upon the culture of colleges, universities, and learned societies. Just as women and non-white social groups were excluded from clerical institutions because they were judged to be susceptible to heresy and heterodoxy – or, at least, to an undiscriminating, incontinent spiritual “enthusiasm” – so scientific elites preserved their power by deploying an epistemology of knowledge that cast “objectivity” as the principal essential attribute of the scientific mind, and members of their own white, male, middle-class fraternity as the most reliable exponents of “objective” observation, experiment, and thought.^66^ The “realm of science,” declared Edwin Hubble in a commencement address to an audience of Caltech graduands in 1938, occupied a distinctive space, sequestered from “the world of values” where the convictions of individuals were shaped by their personal, subjective experiences. The scientist necessarily moved between both worlds, but, whenever he entered the realm of science, he left values behind. In that space, “he attempts to explore the universe as it is, or as it appears to him, uninfluenced by his own desires.”^67^

For the astronomers at Mount Wilson, there existed an additional reason to maintain a vigilant guard against the taint of subjectivity, as many of the methods and instruments they were using, like the 100-inch telescope, were unique to the observatory, and their findings could not be easily checked by researchers at other institutions.^68^ The reputation of the work conducted at Mount Wilson depended upon the reputation of the observatory’s astronomers for policing themselves, and those who could not were off-loaded elsewhere. Hale, for example, assiduously, and without enormous regret, cleared a path for Harlow Shapley, some of whose speculations about the cosmos Hale judged to be “very wild,” to take up a position at Harvard.^69^ For his part, Hubble became increasingly embittered with his Mount Wilson colleague Adriaan van Maanen, whose measurements of rotations in spiral nebulae were discrepant with Hubble’s identification of these nebulae as external to the Milky Way.^70^ By 1935, Hubble wanted to publish evidence that van Maanen was seeing things in his photographic plates that were not there, drawing Adams into months of painful interpersonal diplomacy to keep out of the text Hubble’s insinuation that van Maanen’s observations had been contaminated “by his own desires.”^71^ Hubble also proposed a program of observing time on the 100-inch telescope that gave priority to his concern with galactic distances and recessional velocities, reducing van Maanen’s access to the instrument.^72^ One evening, when van Maanen was scheduled to use the 100-inch, Hubble defied the tradition that the 100-inch observer should sit at the head of the Monastery dining table, moving van Maanen’s napkin to a subordinate place and presiding over the meal himself.^73^

For his part, Adams worried that Hubble’s accomplishments as an astronomer had unduly magnified his self-regard and sense of entitlement, as Hubble frequently abandoned his observational program at Mount Wilson for months at a time in order to travel east and abroad, where he delivered well-recompensed lectures, accepted honorary degrees, and generally took his ease amid the acclaim and hospitality of others.^74^ Hubble and his wife, Grace, suffered from a particularly chronic case of Anglophilia – few things seem to have been more gratifying to them than praise for Hubble’s work murmured across an Oxbridge high table – but their allegiance to the hierarchies and values of interwar Anglo-American social elites was hardly unique within the U.S. astronomical community.^75^ In the first half of the twentieth century, a high proportion of leading American scientists, including astronomers, traced their ancestry back to Puritan England.^76^ Although many centers of physics in the United States, anxious not to be left behind by the revolution in atomic theory taking place in Europe, came to embrace opportunities for intellectual exchange with scholars in and from that revolution’s principal nodes – Copenhagen, Berlin, Göttingen, Leiden, Zurich – American astronomers who had studied on the continent remained minority specimens indeed.^77^ Hale was one of them, which helps to explain his interest in repairing the sinews of transnational scientific cooperation mutilated by the First World War, but his aspirations for Mount Wilson, Caltech, the Huntington Library, and the city of Pasadena reflected a contrasting commitment to an exuberant conception of southern California as the most dynamic contemporary frontier of Anglo-Saxon civilization, as a fertile, sun-kissed environment in which the best of British intellectual and cultural tradition could be nurtured, refreshed, and made ready for the next necessary stage in its march around the globe: the crossing of the Pacific and an encounter with the peoples of Asia.

Broader Anglo-Saxonist ideology, as it washed back and forth through elite British and American circles from the late nineteenth century onward, was septic with strains of racism and racial exceptionalism, and so was its Pasadena subvariant.^78^ Just as Hale projected that the Huntington Library would convert Pasadena into the world’s pre-eminent center for “the study of the history of civilization in Great Britain and America and the sources from which it sprung,” the role of Caltech was to apply the genius of the “English educational system” – which prioritized the selection and training of “men of the highest capacity” – to the auspicious futurity of the Southland, as “the region which is most favorably located for assisting in the solution of the world’s most pressing problems of the next three or four centuries, problems which will have to do with the relations of the eastern and western races.”^79^ Caltech would provide “very exceptional opportunities” for the development of a “relatively small and homogenous” cohort of “very exceptional men”: physically robust, proficient with technology and in the sciences, but also capable of “broad, straight, objective thinking,” informed by the highest cultural and spiritual ideals.^80^ The library and the institute would seed Hale’s cosmology, as it sought both to fathom the imagined depths of Anglo-Saxon political and cultural heritage and to extend the ordering reach of that heritage to the Pacific west and then across the Pacific Ocean itself, with the potential for endless renewal, in the form of intellectual inspiration and the continual propagation of an exclusive fraternity of human talent. Hale had limited patience for anyone whose short-term populist or particularist aspirations threatened to disrupt the consolidation of a world-spanning Anglo-American imperium. As he wrote to his friend the British physicist Sir James Jeans, “in spite of political squabbles and the infernal machinations of Sinn Feiners, Egyptian Nationalists, Indian fakirs, and the unspeakable Hearst, England and the United States must work together and do everything possible to put down the many elements that feed on discord.”^81^

The world that Hale, Adams, and Hubble built around themselves in southern California was structured by protocols of gender, class, and racial containment intended to ensure that the primary spaces of scientific and cultural production in the region were occupied by their own kind: white men, preferably of Yankee stock, with elite educations and a secure standing within bourgeois society. Who else, according to their patrician anthropology, could really be trusted to empty themselves of interest, prejudice, and emotion and, in Hubble’s words, “explore the universe as it is”? In 1910, Hale and the man he had appointed as President of Throop Polytechnic Institute, James Scherer, agreed that women should henceforth be discouraged from applying to the school, thereby quietly establishing a precedent that would transfer to Caltech and keep its undergraduate body all-male into the late 1960s.^82^ The Mount Wilson Observatory, following a practice initiated at Harvard College Observatory in the 1880s, did employ a number of women within its computing division, located at the observatory’s headquarters in downtown Pasadena.^83^ There, the “computers” surveyed the photographic plates of the sun, other stars, and remote nebulae brought down from the mountain by Mount Wilson’s astronomers, calculating distances, rotations, and chemical constitutions, and identifying new objects in the sky; but unlike some of Harvard’s “computers,” they were not encouraged to develop their own research problems and they were only occasionally credited as coauthors in the observatory’s publications.^84^ Mount Wilson – in contrast to Harvard, again, and to the University of California’s Lick Observatory – offered minimal opportunities for women to undertake astronomical observations themselves.^85^ As the observatory expanded its operations on Mount Wilson, the hopes of wilderness experience and masculine renewal that attended its founding had faded, but for decades the summit continued to be a largely homosocial environment; according to Hale, it was simply “impracticable” for women “to take part in observational work” there.^86^ Hale’s prejudices continued to inform observatory policy until the mid-1950s, when the Mount Wilson management, after some huffing and puffing, consented to the British astrophysicist Margaret Burbidge taking over observing runs that it had formally assigned to her husband, Geoffrey.^87^

The observatory drew distinctions between categories of labor with the same rigor as it classified the characteristics of stars.^88^ The female computers received lower salaries than the men who were employed as machinists in the observatory’s workshop on Santa Barbara Street or as technicians on the mountain, and those men, in turn, earned less than Mount Wilson’s “scientific staff.”^89^ Only occasionally did Hale and Adams permit movement across these categories, as in the case of Milton Humason, whose aptitude for photographing and determining the spectra of faint spiral nebulae relieved Hubble from a lot of detailed, tedious work and persuaded an initially doubtful Hale that Humason merited a change in status from night technician to assistant astronomer.^90^ Painful recent experience confirmed Hale and Adams in their preference that those employed at the observatory should know their place. In Mount Wilson’s early years, when the reputation of the observatory depended as much on its prowess in constructing large scientific instruments as on the scope and quality of the actual research it was producing, they had accepted a more porous boundary between the work of the technician and the work of the astronomer. George Ritchey, who designed the observatory’s first large telescope – a sixty-inch reflector – and then spent years in the Santa Barbara Street workshop preparing the primary and secondary mirrors for the 100-inch, was regularly allocated observing time on the sixty-inch to take photographs of nebulae.^91^ But once the 100-inch telescope had entered operation in 1919, Ritchey’s position at Mount Wilson was swiftly and brutally terminated.^92^ Ritchey could certainly be a difficult, temperamental colleague, and Hale and Adams also compiled a list of other “serious defects” in his character, but his dismissal was primarily motivated by a conviction that Ritchey – the son of a cabinetmaker, with his long years of aproned service in the Santa Barbara St. workshop and his side-hustles in growing lemons and making astronomical instruments for a commercial market – no longer had any business commanding the eyepiece of any Mount Wilson telescope now that the observatory was shifting concertedly toward the production of pioneering scientific research.^93^ In the future, Adams noted to Hale, the “value of a man” to the observatory would depend on “his willingness to handle things in a scientific way.” It was, he thought, “hopeless to get Ritchey to see this.”^94^ Adams was looking forward to being “permanently rid of a man who never belonged in scientific work at all.”^95^

Although Mount Wilson had its headquarters in a city that was becoming more racially diverse – in the first two decades of the twentieth century, the Mexican-American, African-American, and Japanese-American communities in Pasadena all increased in size more rapidly than the city’s overall population – the observatory’s scientific operations remained resolutely monocultural.^96^ The validity of a scientific result is conditioned on it staying the same even when the original observer is changed, but the practice at Mount Wilson was not to vary the pool of potential observers very much. Its telescopes may have been constructed in the conviction that advances in knowledge of the cosmos offered a benefit to the whole of humankind, but the social prejudices of Hale and his associates at the observatory and in its surrounding milieu ensured that they could only be accessed by those considered natives of the white Anglo-Saxon or European worlds. Indeed, Hale’s ambitions for Pasadena and its institutions were so exclusive that they could only be advanced by suppressing the opportunities of minority ethnic groups – not just to gain admittance to the field of science but, more generally, to live freely and prosper, participating fully in the affairs of their city.

Hale had been brought up in Chicago, where his family home – and his first purpose-built observatory – had been designed by Burnham and Root, a firm led by the architect Daniel Burnham, a close associate of Hale’s father.^97^ Twenty years later, Hale commissioned Burnham to design the outer tower for Mount Wilson’s 160-foot solar telescope, and then, once that tower had been completed, the dome that would house the 100-inch.^98^ By that point, Burnham had become renowned not just for the individual buildings he had built but also for his broader approach to urban planning, which proposed that all the powerful forces and interests propelling the rapid untamed growth of the fin-de-siècle city could be disciplined and harmonized, via an act of collective will on the part of local elites, to produce a modern American agora composed of uplifting civic buildings and spaces, usually neoclassical in style, served by wide, well-ordered streets and efficient transportation.^99^ Whether in Chicago, Washington, DC, San Francisco, or colonial Manila, social cohesion was to be achieved through a combination of technocratic interventions and inspiring public architecture; there was nothing in Burnham’s approach that required a redistribution of wealth or power from the rich to the poor, nor did it meaningfully address the problem that propertied whites, concerned about African-American migration into their cities, were increasingly as much invested in policing where black people lived as in sublimating racial difference within a vision of the city beautiful.^100^ By the early 1920s, urban planning consultants like Burnham’s protégé, Edward Bennett, had registered the potential market created by these concerns and were exploring how their client cities might use economic zoning ordinances to confine people of color to particular metropolitan neighborhoods, as an alternative to rules that risked the attention of the federal courts by explicitly mandating residential segregation by race.^101^ In 1922, after some initial personal correspondence in which Bennett outlined how a comprehensive civic survey, to include “vital statistics” pertaining to the spatial distribution of different racial groups, could be used to inform both a city and a zoning plan for Pasadena, Hale recommended the consultant’s services to the city’s Board of Directors.^102^ Thereafter, Pasadena’s City Planning Commission, of which Hale was a member, worked with Bennett to develop blueprints for new civic buildings in the center of the city while also enacting ordinances that defined three sizable areas adjacent to the Union Pacific Railroad as either commercial or industrial districts.^103^ The record of the zoning decision did not mention that all three areas were home to concentrated populations of African Americans and Mexican Americans.^104^ The likely intention behind these ordinances was to sharpen the distinction and broaden the economic divide between white and non-white residential neighborhoods. They were a prelude to the restrictive covenants that, with the endorsement of the Board of Directors, were used in the late 1930s and 1940s to prevent homes in white neighborhoods of Pasadena from being sold or rented to “any person not of the white or Caucasian race.”^105^

Hale, Adams, and Hubble all resided in areas of Pasadena that were largely untouched by African-American settlement; the suspicion that they were, on the whole, pretty content with the city’s deepening color line receives no challenge from the records of the Mount Wilson Observatory.^106^ Periodically, during its first decade of operations, the observatory employed Japanese laborers to repair and widen the Mount Wilson trail. Intended to ensure safe passage for the sixty-inch and 100-inch telescopes and all the components required to support them and protect them in place, this work exposed the men carrying it out to significant hazards. In December 1906, one of the laborers, Kakichi Matsushita, fell to his death after the edge of the trail gave way.^107^ Another, identified as “Japanese boy, no. 42,” was injured by falling rocks.^108^ The observatory favored Japanese work gangs because they seemed unusually biddable on the matter of wages and conditions, reflecting Hale’s determination “to prevent any San Francisco labor principles from taking root on Mt. Wilson.”^109^ As one local associate observed to Hale and Adams, “it is remarkable how much they have accomplished for the comparatively small amount of money.”^110^

Hale’s anthropology was not entirely dualistic. He trusted only a relatively narrow category of people to use his telescopes appropriately, while a much broader populace was to be turned away at the gate, but not every exclusion was as absolute as another. Even as a popular movement hostile to Japanese migration became an increasingly powerful force in Californian politics, elite Pasadenans could nurture an appreciation for Japanese art and a wary respect for Japan itself as an advanced world power.^111^ The Hubbles employed a Japanese-American servant to clean their home and wait upon guests.^112^ Hale himself corresponded warmly with astronomers in Tokyo and Kobe.^113^ Jews were another interstitial case, their admission to the scientific community in Pasadena subject to the discretion of those who did not doubt their own right to belong. Antisemitism was commonplace across the circles within which Hale moved, often projecting an apprehension that Jews, if they were afforded unrestricted access to major institutions and professions, would prove even more adept at advancing their collective interests than members of the existing Anglo-American elite. Hale’s brother William, visiting his son, George, at Harvard Law School, reported to Hale that there were “lots of jews” in the classes; George, meanwhile, told his uncle that “the many Jews push the rest of us hard here.”^114^ Hale himself endorsed the continued exclusion of Jews from the private boys’ school to which he had sent his own son.^115^ Robert Millikan, whom Hale had personally recruited to lead the physics faculty at Caltech and to chair the Institute’s executive council, seems to have shared Hale’s prejudices, but both men also recognized that an outright prohibition on the appointment of Jews would cut across their goal of attracting the best scientific talent to Pasadena. They prevaricated, for example, on the question of whether Paul Epstein, a leading authority on quantum theory, should be offered a position to teach theoretical physics, “even though a Jew!”^116^ Eventually, Epstein received his position, while prominent exceptions were also made for Einstein and Albert Michelson, a long-time friend of Hale and former mentor to Millikan at the University of Chicago, who carried out observations with the 100-inch telescope and was also allocated a site on Mount Wilson to conduct an experiment determining the velocity of light. Michelson was Jewish by descent, but not in religious affiliation or, by the lights of contemporary stereotypes, in his cultural style. As Millikan observed approvingly after Michelson’s death, “Neither in aspect nor characteristic did Michelson ever reveal any racial penchants or prejudices. He apparently had no feelings whatever of that sort.”^117^ Those whose identities displayed no tincture of a cosmology alternative to that of “objective” Yankee empiricism, in service to the maintenance of Anglo-Saxon power in the world, could most easily pass as priests into Pasadena’s temples of science.

## The scientific universe as a cosmos?

Hale, Adams, and Hubble would all have resisted the proposition that their commitment to place-making in southern California, concomitant with their belief in the superiority of Anglo-Saxon civilization and the necessity of extending its reach, could be defined as a “cosmology.” Within the scientific precincts of interwar Pasadena, the term “cosmology” was rather strictly reserved for the discussion of astronomical observations, astrophysical dynamics, and relativistic theories relevant to the temporal and spatial totality of the universe. Moreover, white men of status have never found it very easy to accept that they could be the subjects of anthropology. To the extent that Hale, Adams, and Hubble were prepared to identify themselves with a philosophy, it was one that sanctified science as an immaculate world of its own, its methods rigorous, its language austere and exact, and not – at least not primarily – as the means to a social end or as a resource for cultural meaning. Hubble was so anxious to keep his work undefiled by “values” and “desires” that he refused even to presume that his principal scientific achievement – demonstrating the correlation between the distance and outward velocities of galaxies – necessarily meant that the universe was expanding; he was more confident about the “vast scale” of the cosmos, as revealed by his measurements of galactic distance, but remained reticent on the question of its significance for humanity’s sense of self.^118^

Of the three men, it was Hale who was most at ease writing for a popular readership. Early in the career of the Mount Wilson Observatory, he had confidently placed its work in a broader perspective, as part of a multidisciplinary program of research to illuminate the “general scheme of evolution.” For Hale, the program integrated Mount Wilson’s inquiries into the lifecycles of the sun and other stars with the efforts of geologists to probe the history of the earth, with paleontologists and biologists tracking the dance of inheritance and variation in plants and animals, and with anthropologists and historians exploring “the evidences of unity and development in the mental and moral relationships of the peoples of many countries and many generations.”^119^ This was a prospectus, however, underpinned by assumptions – about the scope for effective synthesis across disciplines, about the equivalence of evolution and progress – that would, over time, become bruised and brittle, as new scientific models emerged to perplex the nonspecialist and confound hopes of a universe hospitable to the aspirations of intelligent life.^120^ Even Einstein struggled to commensurate the subatomic dimensions of quantum physics with the amplitudes required for general relativity to work discernible effects. Hale’s brother, William, meanwhile, was perturbed by the prospect that the universe would continue enlarging to the point that most other galaxies would be lost to sight: “I do not quite like to think of the spiral nebulae getting away from us at the great speed which animates them. It is quite unfriendly, – as though they were fleeing before the wrath to come; and we are left alone to tell the tale.”^121^

William had been reading Sir Arthur Eddington’s *The Expanding Universe*, in which Eddington sought to mellow some of the more arresting implications of the contemporary scientific cosmos with analogies drawn from everyday habits and circumstance: a universe that had not always existed was compared with a cut in an office budget, for it now had much less time to accomplish all the tasks allocated to it; the “super-system of galaxies” was “dispersing as a puff of smoke disperses.”^122^ But William was not reassured by Eddington’s genial collapses of spatial and temporal scale, while his brother, in his own popular texts, skirted around the anxieties that they were intended to allay. Hale rarely ventured beyond adept synopses of premodern astronomy and descriptions of contemporary astronomical instruments and methods to explain how the immense, kinetic universe charted by modern science might be experienced as a home, as a nursery of human identity, kinship, and purpose.^123^ It was astronomy, Hale asserted, that had first awakened humans to the laws of nature, so that they had ceased to be “creatures of superstition, surrounded by mysteries, startled at every display of incomprehensible forces” – a statement that suggested a fastidious indifference to the fundamentalist crusades, healing ministries, and esoteric cults fomenting throughout southern California, in the very shadow of Mount Wilson.^124^ Whatever his fellow southlanders were looking for, they were unlikely to find it in the “vast and new conception of an ordered cosmos” that Hale presented as modern astronomy’s gift to the world, a conception that involved “the countless spiral nebulae far beyond our own galactic island, in which the solar system is as a grain of sand.” Within that conception, Hale observed, it was possible to “glimpse the imprint of a Creator, infinitely above the tribal deities of early man, whose immutable laws it is our first duty and greatest advantage to discover and to obey.”^125^ In the moment of composition, Hale may have been able to draw some watery consolation from his invocation of a deistic universe, but his life, taken as a whole, testifies to something else: the attraction of a different cosmology, grounded in the earthly terrain around Mount Wilson, embodied in a tribal hierarchy of human relations, mythic in its imaginings of Anglo-Saxon dominion, past and future, and fashioned through the volitional activity of Hale himself and his acolytes, not the will of a distant watchmaker God inscribed in immutable laws.

## Conclusion

Cosmology is not a term that feels native to the writing of modern history; it is almost certainly used less often than the other words available to modern historians – ideology, power, culture, religion, discourse, network, habitus – as they seek to project some order upon the cluttered, promiscuous carnival of human thought, practice, and association. Within the field of anthropology, in contrast, the frame of cosmology is readily deployed to elucidate features of the contemporary world, even as anthropologists have discarded the assumption that social systems are always integrated at the level of cosmological belief.^126^ Through a reckoning with cosmology, the historian might similarly hope to identify and examine layers of conviction, connection, and concern that remain transparent to the search beams cast by alternative programs of inquiry. When adopted as a tool to think with, cosmology guides our attention to the way that historical actors attributed a direction or pattern to time, imbuing it with meaning and purpose; to the manner in which they conceptualized space, ascribing moral significance to their own position in the context of a larger whole, and organized community in the light of that conception, deciding and then policing who belonged where and who did not belong at all; and to the role played by such temporal and spatial ordering in shielding their world from knowledge of its own contingency, of its vulnerability to change, whether intended by human hand, loosed by arbitrary forces, or disposed by the bias toward entropy within the workings of the wider universe.

To propose that our accounts of modern history might be enriched by thinking about the cosmologies of those we are writing about is not, however, to endorse the thesis of a “return to cosmology” as projected by some anthropologists and philosophers and presumed in many popular science productions and texts. According to this thesis, the scientific study of the cosmos – after a long period of estrangement in the wake of the Copernican and Newtonian revolutions, which stripped away the hierarchies of value that had previously ordered conceptions of the material heavens – has once again become responsive to the concerns of the humanities, and might even serve as a resource for “natural religion” and for shaping the way we live our lives.^127^ Such claims invite a circumspect response. The astronomical and astrophysical achievements of the twentieth century clarified the universe’s history, but not necessarily its purpose. It would take a nihilist or a radical stoic to derive much philosophical sustenance from the most commonly plotted trajectory of a “big bang” universe, with “dark energy” driving galaxies and stars apart from one another and ultimately out toward a cold and lonely death. As the physicist Steven Weinberg famously observed: “The more the universe seems comprehensible, the more it also seems pointless.”^128^

Even for those who constructed the instruments that illuminated the expanding universe, the sort of cosmos that was regarded as most poignant with promise and portent was not always the modern scientific cosmos. For Hale and his colleagues, Mount Wilson represented a fine location to carry out the “new astronomy,” but it was also a setting in which men of their race and class could hope to renew *themselves* through an encounter with wilderness and the Californian sun, learning the ways of the mule as well as the life-courses of the stars. The season in which the astronomers dwelled-in-nature and enjoyed small-group communion on the summit was relatively brief, but it left its mark. Hale remained deeply invested in conceptions of place and community, though the object and field of those investments shifted as the observatory consolidated its presence on the mountain, pulled itself closer to the city of Pasadena, and built its reputation as the scientific world’s premier point of vantage upon a rapidly expanding universe. He now sought to establish Pasadena as an educational and cultural metropole for the men who would ensure that the future was commanded by Anglo-Saxon hands. The quality of the research produced in the city was still important to Hale, but for its own sake less than as a means to an end. The recession of nebulae, tracked on Mount Wilson and explained by Caltech, was not easy to capture within an encouraging cosmology, except as a measure of the city’s significance as a site of intellectual accomplishment. Attuned to the cadence of civilizational time as much as the periods of the stars, Hale created new institutions of knowledge, and a new civic environment around them, in service to his theory of who the knowledge producers were and who they were not. He was fixing a cosmos for himself and his own kind, beneath the runaway skies.

## Footnotes

1. Walter S. Adams, “Early Days at Mount Wilson: Part 2,” *Publications of the Astronomical Society of the Pacific* 59 (1947): 285–304. See also Walter S. Adams, “Early Days at Mount Wilson: Part 1,” *Publications of the Astronomical Society of the Pacific* 59 (1947): 213–31.

2. Albert E. Moyer, *A Scientist’s Voice in American Culture: Simon Newcomb and the Rhetoric of Scientific Method* (Berkeley, CA: University of California Press, 1992), pp.xi, 3, 66–81, 183–201.

3. Adams, “Early Days: Part 2,” 291 (note 1).

4. David Aubin, Charlotte Bigg, and H. Otto Sibum, “Introduction: Observatory Techniques in Nineteenth-Century Science and Society,” in David Aubin, Charlotte Bigg, and H. Otto Sibum (eds.), *The Heavens on Earth; Observatories and Astronomy in Nineteenth-Century Science and Culture* (Durham, NC: Duke University Press, 2010), pp.1–32; Kevin Donnelly, “On the Boredom of Science: Positional Astronomy in the Nineteenth Century,” *British Journal for the History of Science* 47 (2014): 479–503; Moyer, *A Scientist’s Voice*, pp.68–76 (note 2); Donald E. Osterbrock, *Pauper and Prince: Ritchey, Hale, and Big American Telescopes* (Tucson, AZ: University of Arizona Press, 1993), pp.32–6; Simon Schaffer, “Astronomers Mark Time: Discipline and the Personal Equation,” *Science in Context* 2 (1988): 115–45; Albert Van Helden, “Telescope Building, 1850–1900,” in Owen Gingerich (ed.), *The General History of Astronomy, Volume 4: Astrophysics and Twentieth-Century Astronomy to 1950: Part A* (Cambridge: Cambridge University Press, 1984), pp.40–58.

5. George Ellery Hale, “The Development of a New Observatory,” *Publications of the Astronomical Society of the Pacific* 17 (1905): 41–52.

6. Ibid., 2

7. John McAleer, “‘Stargazers at the World’s End’: Telescopes, Observatories and ‘Views’ of Empire in the Nineteenth-Century British Empire,” *British Journal for the History of Science* 46 (2013): 389–413; George Ellery Hale, “The Yerkes Observatory of the University of Chicago: 1. Selection of the Site,” *Astrophysical Journal* 5 (1897): 164–80, 177.

8. K. Maria D. Lane, *Geographies of Mars: Seeing and Knowing the Red Planet* (Chicago, IL: University of Chicago Press, 2011), pp.65–139.

9. Jodicus Wayne Prosser, “Bigger Eyes in the Wider Universe: The American Understanding of Earth in Outer Space, 1893–1941” (PhD dissertation, Texas A&M University, College Station, TX, 2009).

10. John Tresch, “Cosmologies Materialized: History of Science and History of Ideas,” in Darrin M. McMahon and Samuel Moyn (eds.), *Rethinking Modern European Intellectual History* (New York, NY: Oxford University Press, 2014), pp.153–72, 160.

11. ibid., p.163.

12. Alexandre Koyré, *From the Closed World to the Infinite Universe* (New York, NY: Harper and Row, 1958); Michel Blay, *Reasoning with the Infinite: From the Closed World to the Mathematical Universe* (Chicago, IL: University of Chicago Press, 1998); Rémi Brague, *The Wisdom of the World: The Human Experience of the Universe in Western Thought* (Chicago, IL: University of Chicago Press, 2003).

13. Hannah Arendt, *The Human Condition* (Chicago, IL: University of Chicago Press, 1958).

14. Roger D. Launius, “Public Opinion Polls and Perceptions of US Human Spaceflight,” *Space Policy* 19 (3003): 163–75; Robert Poole, *Earthrise: How Man First Saw the Earth* (New Haven, CT: Yale University Press, 2010); Benjamin Lazier, “Earthrise; or, The Globalization of the World Picture,” *American Historical Review* 116 (2011): 602–30; Matthew Tribbe, *No Requiem for the Space Age: The Apollo Moon Landings and American Culture* (New York, NY: Oxford University Press, 2014).

15. Bruno Latour, “Telling Friends from Foes in the Time of the Anthropocene,” in Clive Hamilton, François Gemenne, and Christophe Bonneuil (eds.), *The Anthropocene and the Global Environmental Crisis: Rethinking Modernity in a New Epoch* (London: Routledge, 2015), pp.145–55; Tony Milligan, “Ground Bias: A Driver for Skepticisms about Space Exploration,” *Frontiers in Astronomy and Space Sciences* 9 (2022): 1027251, <www.frontiersin.org/articles/10.3389/fspas.2022.1027251/full> (April 21, 2023).

16. Lisa Messeri, *Placing Outer Space: An Earthly Ethnography of Other Worlds* (Durham, NC: Duke University Press, 2016).

17. Emile Durkheim, *The Elementary Forms of the Religious Life* (London: George Allen & Unwin Ltd, 1915), p.9.

18. Patricia A. Adler, Peter Adler, and Andrea Fontana, “Everyday Life Sociology,” *Annual Review of Sociology* 13 (1987): 217–35.

19. Jeffrey A. Tolbert and Bryan Rupert, “Local Cosmologies,” *Semiotic Review* 8 (2019), <www.semioticreview.com/ojs/index.php/sr/article/view/40/88> (April 21, 2023); Vasudha Narayanan, “Embodied Cosmologies: Sights of Piety, Sites of Power,” *Journal of the American Academy of Religion* 71 (2003): 495–520.

20. See, for example, Lazier, “Earthrise” (note 14); Messeri, *Placing Outer Space*, pp.185–7 (note 16); Gabriela Świtek, “Dasein in a Space Station: The Conquest of Space and the Potentiality of Architecture,” *Montreal Architectural Review* 3 (2016): 69–84.

21. Martin Heidegger, “Building Dwelling Thinking,” in Martin Heidegger, *Poetry, Language, Thought*, trans. into English by Albert Hofstadter (New York, NY: Harper & Row, 1971), pp.143–61, 156; Martin Heidegger, *Being and Time*, trans. into English by John Macquarrie and Edward Robinson (New York, NY: Harper Perennial, 2008), pp.95–122.

22. Richard Wolin, *Heidegger in Ruins: Between Philosophy and Ideology* (New Haven, CT: Yale University Press, 2022).

23. Katy Price, *Loving Faster than Light: Romance and Readers in Einstein’s Universe* (Chicago, IL: University of Chicago Press, 2012).

24. Steven Shapin, “Here and Everywhere: Sociology of Scientific Knowledge,” *Annual Review of Sociology* 21 (1995): 289–321.

25. Jon Agar and Crosbie Smith (eds.), *Making Space for Science: Territorial Themes in the Shaping of Knowledge* (London: Palgrave Macmillan, 1998); Diarmid A. Finnegan, “The Spatial Turn: Geographical Approaches in the History of Science,” *Journal of the History of Biology* 41 (2008): 369–88; Peter Galison and Emily Thompson (eds.), *The Architecture of Science* (Cambridge, MA: MIT Press, 1999); David Livingstone, *Putting Science in Its Place: Geographies of Scientific Knowledge* (Chicago, IL: University of Chicago Press, 2003); Steven Shapin, “Placing the View from Nowhere: Historical and Sociological Problems in the Location of Science,” *Transactions of the Institute of British Geographers* 23 (1998): 5–12.

26. Lane, *Geographies of Mars*, pp.65–95 (note 8); Marjorie Hope Nicholson, *Mountain Gloom and Mountain Glory: The Development of the Aesthetics of the Infinite* (Ithaca, NY: Cornell University Press, 1959).

27. Helen Wright, *Explorer of the Universe: A Biography of George Ellery Hale* (New York, NY: E.P. Dutton, 1966), p.188.

28. Hale to Walcott, July 16, 1929, folder 3, box 42, George Ellery Hale Papers, Caltech Archives (hereafter GEHCA); Wright, *Explorer*, pp.134–78 (note 27).

29. Douglas Brinkley, *The Wilderness Warrior: Theodore Roosevelt and the Crusade for America* (New York, NY: HarperCollins, 2009), pp.502–53.

30. Hale to Adams, March 7, 1904, folder 1, box 2, GEHCA.

31. Adams to Hale, January 28, 1904; Adams to Hale, February 12, 1904; Adams to Hale, February 25, 1904: folder 1, box 2, GEHCA.

32. Wright, *Explorer*, pp.185–96 (note 27).

33. Adams, “Early Days: Part 1,” 222 (note 1); Adams, “Early Days: Part 2,” (note 1).

34. Robert Curzon, *Visits to the Monasteries of the Levant* (London: Jon Murray, 1851), pp.52–6.

35. Adams, “Early Days: Part 2,” 288 (note 1).

36. George E. Hale, *Ten Years’ Work of a Mountain Observatory: A Brief Account of the Mount Wilson Solar Observatory of the Carnegie Institution of Washington* (Washington, DC: Carnegie Institution of Washington, 1915).

37. David H. DeVorkin, “The Discovery and Exploitation of Spectroscopic Parallaxes,” in A. G. David Philip, W. F. van Altena, and A. R. Upgren (eds.), *Anni Mirabiles: A Symposium Celebrating the 90th Birthday of Dorrit Hoffleit* (Schenectady, NY: L. David Press, 1999), pp.17–23.

38. George E. Hale, “Report on the Solar Observatory, Mount Wilson, California,” in *Carnegie Institution of Washington Year Book No. 5, 1906* (Washington, DC: Carnegie Institution of Washington, 1907), pp.60–86, 62–3.

39. George E. Hale, “Mount Wilson Solar Observatory,” in *Carnegie Institution of Washington Year Book No. 12, 1913* (Washington, DC: Carnegie Institution of Washington, 1914), pp.194–240, 232–3.

40. Hale to Staats, August 4, 1909, folder 14, box 168, George Ellery Hale Papers, Huntington Library (hereafter GEHHL); Adams, “Early Days: Part 2,” 292 (note 1).

41. Ritchey to Union Iron Works, August 22, 1905, folder 10, box 170, GEHHL; Hale to Jouvard, April 4, 1907, folder 13, box 155, GEHHL; Mount Wilson Solar Observatory to Fore River Shipbuilding Company, June 11, 1913, folder 1, box 155, GEHHL.

42. George E. Hale, “Report of Director of the Solar Observatory, Mount Wilson, California,” in *Carnegie Institution of Washington Year Book No. 4, 1905* (Washington, DC: Carnegie Institution of Washington, 1906), pp.56–77, 76–7; George E. Hale, “Mount Wilson Solar Observatory,” in *Carnegie Institution of Washington Year Book No. 11, 1912* (Washington, DC: Carnegie Institution of Washington, 1913), pp.172–213, 211–13.

43. Adams, “Early Days: Part 2,” 289 (note 1).

44. Adams, “Early Days: Part 1,” 221–2 (note 1).

45. Adams to Merriam, November 10, 1924, folder: “Merriam, John C., 1924 Jul-Dec,” box 41, Papers of Walter S. Adams, Huntington Library (hereafter WSAHL).

46. Walter Adams, “Biographical Notes on Walter S. Adams,” August 1943; Walter Adams, “Biographical Notes – Walter S. Adams,” March 1954: Deceased Members Files, National Academy of Sciences.

47. Adams to Fox, September 11, 1934, folder: “Griffith Observatory 1933–1943,” box 32, WSAHL; Kevin Starr, *Endangered Dreams: The Great Depression in California* (New York, NY: Oxford University Press, 1996), pp.121–55; Greg Mitchell, *The Campaign of the Century: Upton Sinclair’s Race for Governor of California and the Birth of Media Politics* (New York, NY: Random House, 1992).

48. William Sheehan and Donald E. Osterbrock, “Hale’s ‘Little Elf’: The Mental Breakdowns of George Ellery Hale,” *Journal for the History of Astronomy* 31 (2000): 93–114.

49. Osterbrock, *Pauper and Prince*, pp.218–9 (note 4).

50. Hale to Scherer, May 9, 1908, folder 6, box 36, GEHCA; Judith R. Goodstein, *Millikan’s School: A History of the California Institute of Technology* (New York, NY: Norton, 1991), pp.30–87; Wright, *Explorer*, pp.237–51 (note 27).

51. Hale, “Autobiographical Notes,” February 8, 1933, folder 1, box 92, GEHCA, pp. 28–31; Wright, *Explorer*, pp.371–86 (note 27); James Thorpe, *Henry Edwards Huntington: A Biography* (Berkeley, CA: University of California Press, 1994), pp.386–93.

52. Hale to Patton, April 11, 1925, folder 2, box 8, GEHCA.

53. Kevin Starr, *Inventing the Dream: California through the Progressive Era* (New York, NY: Oxford University Press, 1985), pp.64–98.

54. Hale, “Art and Environment,” lecture to Pasadena Music and Art Association, February 27, 1914, folder 1, box 73, GEHCA; Hale to Huntington, May 11, 1914, folder 17, box 22, GEHCA; Hale, “Autobiographical Notes,” February 8, 1933, folder 1, box 92, GEHCA, p.31; Hale to Merrill, October 3, 1927, folder 10, box 11, GEHCA; Hale to Smithells, March 10, 1931, folder 20, box 38, GEHCA; William S. Barton, “Athens Rebuilt in the West,” *Los Angeles Times*, December 10, 1933.

55. George Ellery Hale, “War Services of the National Research Council,” in Robert M. Yerkes (ed.), *The New World of Science* (New York, NY: The Century Co., 1920), pp.13–30; Wright, *Explorer*, pp.285–306 (note 27).

56. Hale to Pickering, August 9, 1918; Preamble to Resolutions Adopted by Inter-Allied Conference on International Scientific Organizations, October 1918; Hale to Wilson, October 15, 1918: folder 1, box 47, GEHCA. On Hale’s international outlook, see David DeVorkin, “George Ellery Hale’s Internationalism,” in Christiaan Sterken, John Hearnshaw, and David Valls-Gabaud (eds.), *Under One Sky: The IAU Centenary Symposium (IAU S349): Proceedings of the International Astronomical Union* (Cambridge: Cambridge University Press, 2019), pp.153–60.

57. Robert W. Smith, *The Expanding Universe: Astronomy’s ‘Great Debate’ 1900–1931* (Cambridge: Cambridge University Press, 1982), pp.111–19.

58. Ibid., pp.180–93.

59. Jeffrey Crelinsten, *Einstein’s Jury: The Race to Test Relativity* (Princeton, NJ: Princeton University Press, 2006), pp.315–20.

60. Adams, “Professor Einstein’s Visit to Pasadena and the Mount Wilson Observatory,” typescript, c. 1931, folder: “Manuscript: Albert Einstein [1931],” box 105, WSAHL; Harry Nussbaumer, “Einstein’s Conversion from His Static to an Expanding Universe,” *European Physical Journal H* 39 (2014): 37–62; “Einstein Gives View of Man and Science,” *New York Times*, February 3, 1931.

61. “Einstein Stumps Scientists,” *Los Angeles Times*, January 15, 1931.

62. Hale to Einstein, February 26, 1931, folder 13, box 15, GEHCA.

63. Lee Shippey, “Leeside o’ L.A.: Personal Glimpses of Famous Southlanders,” *Los Angeles Times*, January 4, 1931.

64. Mircea Eliade, *The Sacred and Profane: The Nature of Religion* (New York, NY: Harcourt, Inc., 1959), pp.20–4.

65. C. Scott Littleton, *The New Comparative Mythology: An Anthropological Assessment of the Theories of Georges Dumézil* (Berkeley, CA: University of California Press, 1966).

66. Lorraine Code, *What Can She Know? Feminist Theory and the Construction of Knowledge* (Ithaca, NY: Cornell University Press, 1991); Sandra Harding, *Whose Science? Whose Knowledge? Thinking from Women’s Lives* (Ithaca, NY: Cornell University Press, 1991); Evelyn Fox Keller, *Reflections on Gender and Science* (New Haven, CT: Yale University Press, 1995), pp.75–94; David F. Noble, *A World Without Women: The Christian Clerical Culture of Western Science* (New York, NY: Alfred A. Knopf, 1992); Ann Taves, *Fits, Trances, and Visions: Experiencing Religion and Explaining Experience from Wesley to James* (Princeton, NJ: Princeton University Press, 1999).

67. Hubble, “Commencement Address to the California Institute of Technology: Experiment and Experience,” June 10, 1938, folder 23, box 1, Papers of Edwin Powell Hubble, Huntington Library (hereafter EPHHL).

68. Hale, *Ten Years’ Work*, pp.4–6 (note 36).

69. Hale to Shapley, February 27, 1919, folder 6, box 168, GEHHL; Lowell to Hale, February 27, 1920, folder 3, box 27, GEHCA; Hale to Lowell, March 12, 1920, folder 3, box 27, GEHCA; Hale to Lowell, March 20, 1920, folder 3, box 27, GEHCA; Hale to Lowell, December 11, 1920, folder 6, box 168, GEHHL.

70. Smith, *The Expanding Universe*, pp.131–6 (note 57); Gale E. Christianson, *Edwin Hubble: Mariner of the Nebulae* (Bristol: Institute of Physics Publishing, 1995), pp.230–4.

71. Seares to Hale, January 24, 1935, folder 2, box 38, GEHCA; Seares to Hale, January 25, 1935, folder 2, box 38, GEHCA; Adams to Merriam, “Confidential Statement for President Merriam,” August 15, 1935, folder: “Hubble Edwin Powell 1921-1935,” box 34, WSAHL.

72. Hubble to Adams, October 30, 1934, with attached “Memorandum on the 100-inch Program for the Dark of the Moon”; Adriaan van Maanen, undated handwritten note, c. October 1934: folder: “Hubble Edwin Powell 1921-1935,” box 34, WSAHL.

73. Interview of Olin Wilson by David DeVorkin on July 11, 1978, Niels Bohr Library & Archives, American Institute of Physics (hereafter NBLA), <www.aip.org/history-programs/niels-bohr-library/oral-histories/4315> (April 21, 2023).

74. Adams to Merriam, September 30, 1935; Adams to Merriam, November 22, 1935; Adams to Merriam, February 19, 1936; folder: “Hubble Edwin Powell 1921-1935,” box 34, WSAHL. Also Grace Hubble, “Travels, 1934,” folder 74, box 4, EPHHL; Grace Hubble, “Travel Diary,” 1936, folder 75, box 5, EPHHL.

75. Christianson, *Edwin Hubble*, pp.249–58 (note 70).

76. Stephen Sargent Visher, *Scientists Starred, 1903–1943, in* *“American Men of Science”: A Study of Collegiate and Doctoral Training, Birthplace, Distribution, Backgrounds, and Developmental Influences* (Baltimore, MD: Johns Hopkins University Press, 1947), pp.102–4, 426.

77. Stanley Coben, “The Scientific Establishment and the Transmission of Quantum Mechanics to the United States, 1919–32,” *American Historical Review* 76 (1971): 442–66; Daniel J. Kevles, *The Physicists: The History of a Scientific Community in Modern America* (Cambridge, MA: Harvard University Press, 1995), pp.200–21; John Lankford, *American Astronomy: Community, Careers, and Power, 1859-1940* (Chicago, IL: University of Chicago Press, 1997), pp.84–5.

78. John Higham, *Strangers in the Land: Patterns of American Nativism, 1860–1925* (New Brunswick, NJ: Rutgers University Press, 1955), pp.131–57; Gail Bederman, *Manliness and Civilization: A Cultural History of Gender and Race in the United States, 1880–1917* (Chicago, IL: University of Chicago Press, 1995); Paul A. Kramer, “Empire, Exceptions, and Anglo-Saxons: Race and Rule between the British and United States Empires, 1880–1910,” *Journal of American History* 88 (2002): 1315–53.

79. Hale to Patton, April 11, 1925, folder 2, box 8, GEHCA; “California Institute of Technology: Its Direction, Aims, Accomplishments, Needs and Financial Condition,” undated (c. 1923), folder 20, box 6, GEHCA. Robert Kargon identifies Robert Millikan, whom Hale selected to chair the Institute’s Executive Council, as the author of this document, but notes its continuity with Hale’s own themes. Robert H. Kargon, “Temple to Science: Cooperative Research and the Birth of the California Institute of Technology,” *Historical Studies in the Physical Sciences* 8 (1977): 3–31, 18.

80. “California Institute of Technology: Its Direction, Aims, Accomplishments, Needs and Financial Condition,” undated (c. 1923), folder 20, box 6, GEHCA.

81. Hale to Jeans, May 25, 1920, folder 12, box 23, GEHCA.

82. Scherer to Hale, September 26, 1910, folder 6, box 36, GEHCA; Goodstein, *Millikan’s School*, p.31 (note 50); Amy Sue Bix, *Girls Coming to Tech! A History of American Engineering Education for Women* (Cambridge, MA: MIT Press, 2013), pp.191–213.

83. Dava Sobel, *The Glass Universe: The Hidden History of the Women Who Took the Measure of the Stars* (London: 4th Estate, 2017), pp.8–14.

84. Lankford, *American Astronomy*, pp.342–7 (note 77); Eun-Joo Ahn, “Finding the Invisible Workers in Astronomy: The Case of Mount Wilson Observatory,” *Historical Studies in the Natural Sciences* 52 (2022): 555–88.

85. Sobel, *Glass Universe*, p.75 (note 83); Sheila M. Humphreys, “Celestial Observers: First Sixteen Berkeley Women Doctoral Graduates in Astronomy 1913–1952,” University of California Berkeley Chancellor’s Immediate Office, *150 Years of Women at Berkeley*, 2020, <https://150w.berkeley.edu/celestial-observers-first-sixteen-berkeley-women-doctoral-graduates-astronomy-1913-1952> (May 30, 2022); Robert S. Richardson, “Astronomy: The Distaff Side,” *Astronomical Society of the Pacific Leaflet* 4, no. 18 (1944): 238. Eun-Joo Ahn notes two short-term exceptions to Mount Wilson’s men-only observing regime during the first twenty years of the observatory’s operations: Jennie B. Lasby and Margaret Harwood. Ahn, “Finding the Invisible Workers in Astronomy,” 580–1 (note 84).

86. Hale to Pettit, August 5, 1920, folder 19, box 164, GEHHL.

87. Ahn, “Finding the Invisible Workers in Astronomy,” 580 (note 84); interview of E. Margaret Burbidge by David DeVorkin on July 13, 1978, NBLA, <www.aip.org/history-programs/niels-bohr-library/oral-histories/25487> (April 21, 2023).

88. Ahn, “Finding the Invisible Workers in Astronomy” (note 84).

89. Adams, “Mount Wilson Observatory: Estimated Expenses for 1925,” enclosed with Adams to Hale, September 2, 1924, folder 1, box 2, GEHCA.

90. Christianson, *Edwin Hubble*, pp.185–90 (note 70); Ronald L. Voller, *The Muleskinner and the Stars: The Life and Times of Milton La Salle Humason, Astronomer* (New York, NY: Springer, 2016), pp.99–134.

91. Osterbrock, *Pauper and Prince*, pp.66–8, 79–102, 108–9, 139, 142–7 (note 4).

92. Ibid., pp.154–62.

93. Hale to Woodward, August 19, 1919, folder 9, box 44, GEHCA; Adams to Hale, February 2, 1919, folder 3, box 148, GEHHL.

94. Adams to Hale, February 2, 1919, folder 3, box 148, GEHHL.

95. Adams to Hale, December 8, 1918, folder 3, box 148, GEHHL.

96. On the racial and ethnic history of Pasadena, see Carson Anderson, “Ethnic History Research Project: Pasadena, California: Report of Survey Findings,” March 1995, <http://pasadena.cfwebtools.com/images/other/EthnicHistoryResearchReport(1995Version).pdf> (July 5, 2022); James E. Crimi, “The Social Status of the Negro in Pasadena, California,” (MA thesis, University of Southern California, Los Angeles, CA, June 1941).

97. Wright, *Explorer*, p.54 (note 27); Osterbrock, *Pauper and Prince*, p.24 (note 4).

98. Hale to Burnham, January 27, 1909; Adams to Burnham, October 18, 1911: folder 7, box 151, GEHHL.

99. Thomas S. Hines, *Burnham of Chicago: Architect and Planner* (Chicago, IL: University of Chicago Press, 1974); Carl Smith, *The Plan of Chicago: Daniel Burnham and the Remaking of the American City* (Chicago, IL: University of Chicago Press, 2006).

100. Rebecca Tinio McKenna, *American Imperial Pastoral: The Architecture of US Colonialism in the Philippines* (Chicago. IL: University of Chicago Press, 2017), pp.75–110; Margaret Garb, “Race, Housing and Burnham’s Plan: Why Is There No Housing in the 1909 Plan of Chicago?” *Journal of Planning History* 10 (2001): 99–113.

101. Richard Rothstein, *The Color of Law: A Forgotten History of How Our Government Segregated America* (New York, NY: Liveright, 2017), pp.43–54.

102. Bennett to Hale, February 1, 1922, including “City of Pasadena: General Memorandum on a Plan” and “Memorandum and Estimate: Outlining the Scope of a City Plan and Zoning Plan for the City of Pasadena, California,” folder 10, box 11, GEHCA; Hale to Hoffmann, July 7, 1925, folder 8, box 74, GEHCA.

103. French to Hale, January 21, 1923; Halsted to City of Pasadena Planning Commission, October 2, 1924: folder 10, box 11, GEHCA.

104. Crimi, “Social Status,” pp.15–19 (note 96).

105. Michael E. James, *The Conspiracy of the Good: Civil Rights and the Struggle for Community in Two American Cities, 1875–2000* (New York, NY: Peter Lang, 2005), p.248.

106. 1930 U.S. federal census records cross-referenced with map contained in Crimi, “Social Status,” p.16 (note 96).

107. Simons to Hale, December 14, 1906, folder 7, box 168, GEHHL.

108. Hale to Talbott, May 27, 1908; Talbott to Carnegie Institution of Washington, Pasadena, June 1, 1908: folder 12, box 159, GEHHL.

109. Hale to McGregor, July 31, 1907, folder 10, box 170, GEHHL.

110. Staats to Mount Wilson Solar Observatory, September 17, 1913, folder 14, box 168, GEHHL.

111. Roger Daniels, *The Politics of Prejudice: The Anti-Japanese Movement in California and the Struggle for Japanese Exclusion* (Berkeley, CA: University of California Press, 1977); Kevin Starr, *Material Dreams: Southern California through the 1920s* (New York, NY: Oxford University Press, 1990), pp.189–91.

112. Christianson, *Edwin Hubble*, p.197 (note 70).

113. Hale to Kikuchi, March 7, 1908, folder 11, box 25, GEHCA; Hale to Sekiguti, April 9, 1926, folder 3, box 38, GEHCA.

114. William Hale to George Ellery Hale, February 22, 1936; George Ellery Hale II to George Ellery Hale, November 3, 1935: folder 1, box 20, GEHCA.

115. Thacher to Hale, December 3, 1924; Thacher to Hale, December 16, 1924: folder 7, box 40, GEHCA.

116. Millikan to Hale, July 16, 1921; Hale to Millikan, July 26, 1921; Milliken to Hale, telegram, July 26, 1921; Milliken to Hale, August 6, 1921: folder 2, box 29, GEHCA.

117. Robert A. Millikan, “Biographical Memoir of Albert Abraham Michelson 1852–1931,” in *National Academy of Sciences Biographical Memoirs: Volume 19* (Washington, DC: National Academy of Sciences, 1938), pp.121–46, 128–9.

118. Edwin Hubble, *The Realm of the Nebulae* (London: Oxford University Press, 1936), pp.1–2, 198–202; Hubble, “The Exploration of Space,” typescript of talk delivered at the Carnegie Institution of Washington, winter 1928–9, folder 24, box 1, EPHHL.

119. George Ellery Hale, *The Study of Stellar Evolution: An Account of Some Recent Methods of Astrophysical Research* (Chicago, IL: University of Chicago Press, 1908), pp.1–5.

120. Nasser Zakariya, *A Final Story: Science, Myth, and Beginnings* (Chicago, IL: Chicago University Press, 2017), pp.145–79.

121. William B. Hale to George Ellery Hale, March 5, 1933, folder 1, box 20, GEHCA.

122. Sir Arthur Eddington, *The Expanding Universe* (Cambridge: Cambridge University Press, 1933), pp.13, 85; Price, *Loving Faster than Light*, pp.100–27 (note 23).

123. George Ellery Hale, *The New Heavens* (New York, NY: Charles Scribner’s Sons, 1922); George Ellery Hale, *The Depths of the Universe* (New York, NY: Charles Scribner’s Sons, 1924); George Ellery Hale, *Beyond the Milky Way* (New York, NY: Charles Scribner’s Sons, 1926); George Ellery Hale, *Signals from the Stars* (London: Charles Scribner’s Sons, 1932).

124. Hale, *Signals*, pp.133–4 (note 123); Starr, *Material Dreams*, pp.131–44 (note 111); Matthew Avery Sutton, *Aimee Semple McPherson and the Resurrection of Christian America* (Cambridge, MA: Harvard University Press, 2007); Carey McWilliams, “Mecca of the Miraculous,” in Dean Stewart and Jeannine Gendar (eds.), *Fool’s Paradise: A Carey McWilliams Reader* (Santa Clara, CA: Santa Clara University, 2001), pp.17–29.

125. Hale, *Signals*, p.137 (note 123).

126. Allen Abramson and Martin Holbraad (eds.), *Framing Cosmologies: The Anthropology of Worlds* (Manchester: Manchester University Press, 2014).

127. Koyré, *From the Closed World* (note 12); Stephen Toulmin, *The Return to Cosmology: Postmodern Science and the Theology of Nature* (Berkeley, CA: University of California Press, 1982); Allen Abramson and Martin Holbraad, “The Cosmological Frame in Anthropology,” in Allen Abramson and Martin Holbraad, *Framing Cosmologies: The Anthropology of Worlds* (Manchester: Manchester University Press, 2014), pp.1–28, 10–15; Gregory Schrempp, *The Ancient Mythology of Modern Science: The Ancient Mythology of Modern Science: A Mythologist Looks (Seriously) at Popular Science Writing* (Montreal, Canada: McGill-Queen’s University Press, 2012), pp.222–32.

128. Steven Weinberg, *The First Three Minutes: A Modern View of the Origin of the Universe* (New York, NY: Basic Books, 1977), p.154.

